# Safety and hemodynamic efficacy of the LVIS stent in the endovascular treatment of intracranial wide-necked aneurysms: a single-center retrospective study

**DOI:** 10.1186/s41016-026-00435-9

**Published:** 2026-06-29

**Authors:** Bowen Dong, Xiang Xu, Dayong Wang, Jian Pei, Yu Zheng, Yun He Gao, Zhaoguo Gao

**Affiliations:** Department of Neurosurgery, Tangshan Gongren Hospital, Tangshan, 063000 China

**Keywords:** LVIS stent, Intracranial aneurysm, Wide-necked aneurysm, Stent-assisted coiling, Hemodynamics, Computational fluid dynamics

## Abstract

**Background:**

Treating wide-necked aneurysms endovascularly is still difficult. Because of its high metal coverage and flow-diverting capabilities, the LVIS stent may lead to better results. We carried out this retrospective analysis to assess its hemodynamic impact, short-term efficacy, and procedural safety.

**Methods:**

An observational, exploratory retrospective study was conducted between January and December 2023 on 61 consecutive patients with intracranial wide-necked aneurysms receiving LVIS stent-assisted coiling. The group consisted of 35 patients with ruptured aneurysms and 26 people with unruptured aneurysms. The Raymond-Roy Occlusion Classification (RROC) was used to assess the initial angiographic data. At 3 to 6 months, clinical outcomes were evaluated using the modified Rankin Scale (mRS), and parent artery patency and aneurysm recurrence were evaluated using follow-up digital subtraction angiography (DSA).

Furthermore, a subset of patients underwent computational fluid dynamics (CFD) analysis to measure changes in intra-aneurysmal mean flow velocity and wall shear stress (WSS) in order to quantify hemodynamic modifications.

**Results:**

This study included 35 patients with ruptured and 26 with unruptured aneurysms, with 61 patients totally. Immediate complete occlusion (RROC class I) was achieved in 80.0% (28/35) of ruptured and 84.6% (22/26) of unruptured aneurysms. Angiographic follow-up (available for 27 patients, 44.3%) at 6–12 months demonstrated stable occlusion in all initial RROC-I cases. Furthermore, in the ruptured cohort, the rate of favorable outcome (mRS 0–1) increased from 57.1% at discharge to 77.1% at follow-up, while 96.2% of unruptured patients maintained an mRS of 0–1. Procedure-related complications involved intraprocedural thrombosis (2 ruptured cases) and one fatal in-hospital stent thrombosis (unruptured case), resulting in overall morbidity and mortality rates of 16.4% and 1.6%, respectively. CFD analysis demonstrated significant and durable hemodynamic improvements. Specifically, post-operative reductions in relative inflow, aneurysm volume with high flow, and WSS were all sustained at 6-month follow-up (all *P* < 0.001).

**Conclusions:**

Our study provides preliminary evidence that LVIS stent-assisted coiling may be related to high aneurysm occlusion rate, functional recovery in ruptured cases, and favorable hemodynamic modification. However, this study fails to achieve causal inference due to the retrospective design, and the complication profile underscores procedural risks, highlighting the need for prospective, comparative studies to confirm its safety and efficacy profile.

## Background

Localized sac-like dilations caused by localized weakening and bulging of the cerebral artery wall are known as intracranial aneurysms. Aneurysm ruptures pose a major risk to public health because they can cause subarachnoid hemorrhage, which can result in significant disability and death. Endovascular treatment is now recognized as the main therapeutic option for intracranial aneurysms due to advancements in imaging technology and ongoing development of neurointerventional devices.

Wide-necked aneurysms, however, provide unique technical difficulties (neck width > 4 mm or dome-to-neck ratio < 2). Because the neck is not well defined, the coil may move the parent artery during embolization. In this situation, stent-assisted coiling has become a game-changing strategy. By using this method, a stent can be placed across the aneurysm neck to act as a scaffold. This could improve packing density and procedural safety by strengthening the coil stability inside the aneurysm sac.

A more recent braided, self-expanding nitinol device is the low-profile visualized intraluminal support (LVIS) stent. Because of their high metal coverage, superior wall apposition, and complete resheathability, these stents may theoretically aid in flow diversion and encourage intra-aneurysmal thrombosis.

Previous research has demonstrated the technical viability and short-term anatomical success of LVIS-assisted embolization. However, a thorough evaluation of its safety profile is still lacking, especially with regard to its direct effects on aneurysm hemodynamics, which calls for additional quantitative confirmation. Crucially, computational fluid dynamics (CFD) is a potent tool for simulating and measuring hemodynamic parameters including intra-aneurysmal flow velocity and wall shear stress (WSS), providing new insights into the effectiveness of treatment. Furthermore, it is becoming more widely acknowledged that endovascular treatments lower the long-term risk of aneurysm recurrence through hemodynamic alteration.

In light of the aforementioned, a series of wide-necked cerebral aneurysms were used in this retrospective study to thoroughly investigate procedural safety of LVIS-assisted coiling by evaluating complication rates and clinical outcomes. Additionally, this study used CFD analysis to quantitatively examine hemodynamic changes prior to and following intervention in order to assess the therapeutic efficacy from a biomechanical standpoint. It is anticipated that this combined strategy would yield stronger evidence supporting the clinical application of LVIS stents.

## Methods

### Patient cohort and treatment decision pathway

For ruptured aneurysms, emergency assessment determined whether primary coiling was technically feasible. When simple coiling was possible, the double-microcatheter technique was optionally employed to enhance coil stability and achieve adequate packing density. When simple coiling was not feasible due to complex neck anatomy or sac morphology, stent-assisted coiling (SAC) was employed. Balloon-assisted coiling (BAC) was not routinely performed at our center, as SAC provides equivalent or superior mechanical scaffolding and promotes intra-aneurysmal flow remodeling, consistent with published evidence.

For unruptured aneurysms with a high projected recurrence risk (e.g., largeyin size, complex shape), SAC was considered the primary strategy. Intrasaccular flow-diverting devices were not available during the study period and therefore were not performed. Microsurgical clipping remains a potential alternative; however, the present discussion focuses on endovascular treatment strategies.

In both ruptured and unruptured aneurysms meeting these criteria, the LVIS stent was selected for SAC to provide immediate mechanical scaffolding, enhance long-term aneurysm healing through flow remodeling, and maintain a favorable procedural safety profile. Other widely reported techniques, including BAC, intrasaccular devices, flow diverters, or alternative stent designs, were not routinely utilized at our institution.

### Baseline clinical and anatomical characteristics

We included 61 consecutive patients with intracranial wide-necked aneurysms undergoing LVIS stent-assisted coiling between January and December 2023 in this single-center, retrospective research. The cohort comprised 26 individuals with unruptured aneurysms (the unruptured aneurysm group) and 35 patients with acutely ruptured aneurysms (the ruptured aneurysm group).

Acute symptoms included severe headache, nausea/vomiting, neurological impairments, changed consciousness, and seizures. The group with ruptured aneurysms consisted of 31 females and 4 males, with a mean age of 60.17 ± 9.24 years. Due to severe hemorrhagic presentation, this group’s pre-treatment mRS scores varied from 0 to 4, including 5 patients at mRS 0, 18 patients at mRS 1, 9 patients at mRS 2, and 3 patients at mRS 3.

The mean age of the 22 females and 4 men in the unruptured aneurysm group was 57.4 ± 8.76 years. Two individuals had oculomotor nerve palsy, and one patient was receiving stepwise treatment for a right ophthalmic segment aneurysm after a previous rupture. The majority of patients (88.5%, 23/26) were asymptomatic incidental discoveries. With 23 patients at mRS 0 and 3 patients at mRS 1, the pre-treatment mRS scores in this group were primarily 0–1, indicating retained baseline neurological function.

Within 72 h of the onset of symptoms or a conclusive diagnosis, all patients received pertinent procedures. A clear reference for assessing post-procedural functional outcomes is provided by including these pre-treatment mRS data (Table 1).

**Table 1:** Initial features of individuals with ruptured and unruptured intracranial aneurysms

Characteristic	Unruptured aneurysms (*n* = 26)	Ruptured aneurysms (*n* = 35)
Demographics		
Female, *n* (%)	22(84.6%)	31 (88.5%)
Mean age (years)	57.4	60.17
Aneurysm location, *n* (%)		
Posterior communicating artery	7 (26.9%)	29 (82.9%)
Ophthalmic segment	18 (69.2%)	3 (8.6%)
Vertebral artery (V4)	1 (3.8%)	-
Cavernous sinus segment	-	1 (2.9%)
Posterior circulation	-	2 (5.7%)
Aneurysm size, *n* (%)		
Small (< 5 mm)	11 (42.3%)	18 (51.4%)
Medium (5–10 mm)	9 (34.6%)	12 (34.3%)
Large (10–25 mm)	6 (23.1%)	5 (14.3%)
Clinical presentation, *n* (%)		
Incidental finding	23 (88.5%)	-
Oculomotor nerve palsy	2 (7.7%)	-
Hunt-Hess Grade	-	
Grade 1	-	5 (14.3%)
Grade 2	-	18 (51.4%)
Grade 3	-	9 (25.7%)
Grade 4	-	3 (8.6%)
Pre-treatment mRS		
0	23	5
1	3	18
2	0	9
3	0	3
4	0	0
discharge mRS score, *n* (%)		
0	23 (88.5%)	0
1	2 (7.7%)	20 (57.1%)
2	0	5 (14.3%)
3	0	4 (11.4%)
4	0	3 (8.6%)
5	0	3 (8.6%)
6	1 (3.8%)	0
WFNS Grade		
I	-	5 (14.3%)
II	-	18 (51.4%)
III	-	9 (11.4%)
IV	-	3 (8.6%)
V	-	0
Treatment history, *n* (%)		
Staged surgery (Stage II)	1 (3.8%)	-

### Imaging protocol and aneurysm characterization

#### Diagnostic imaging workflow

A uniform imaging approach was used to assess each patient. In particular, people who presented with clinical indications suggestive of acute rupture had urgent non-contrast cranial computed tomography (CT) to establish the existence of subarachnoid hemorrhage. In the meanwhile, cases with unruptured aneurysms were first identified using either magnetic resonance angiography (MRA) or cranial computed tomography angiography. For every patient, digital subtraction angiography (DSA) was the gold-standard diagnostic technique. Based on established morphometric criteria (neck width ≥ 4 mm and/or a dome-to-neck ratio ≤ 2), it would make it easier to definitively confirm wide-necked saccular aneurysms.

**Fig. 1:**
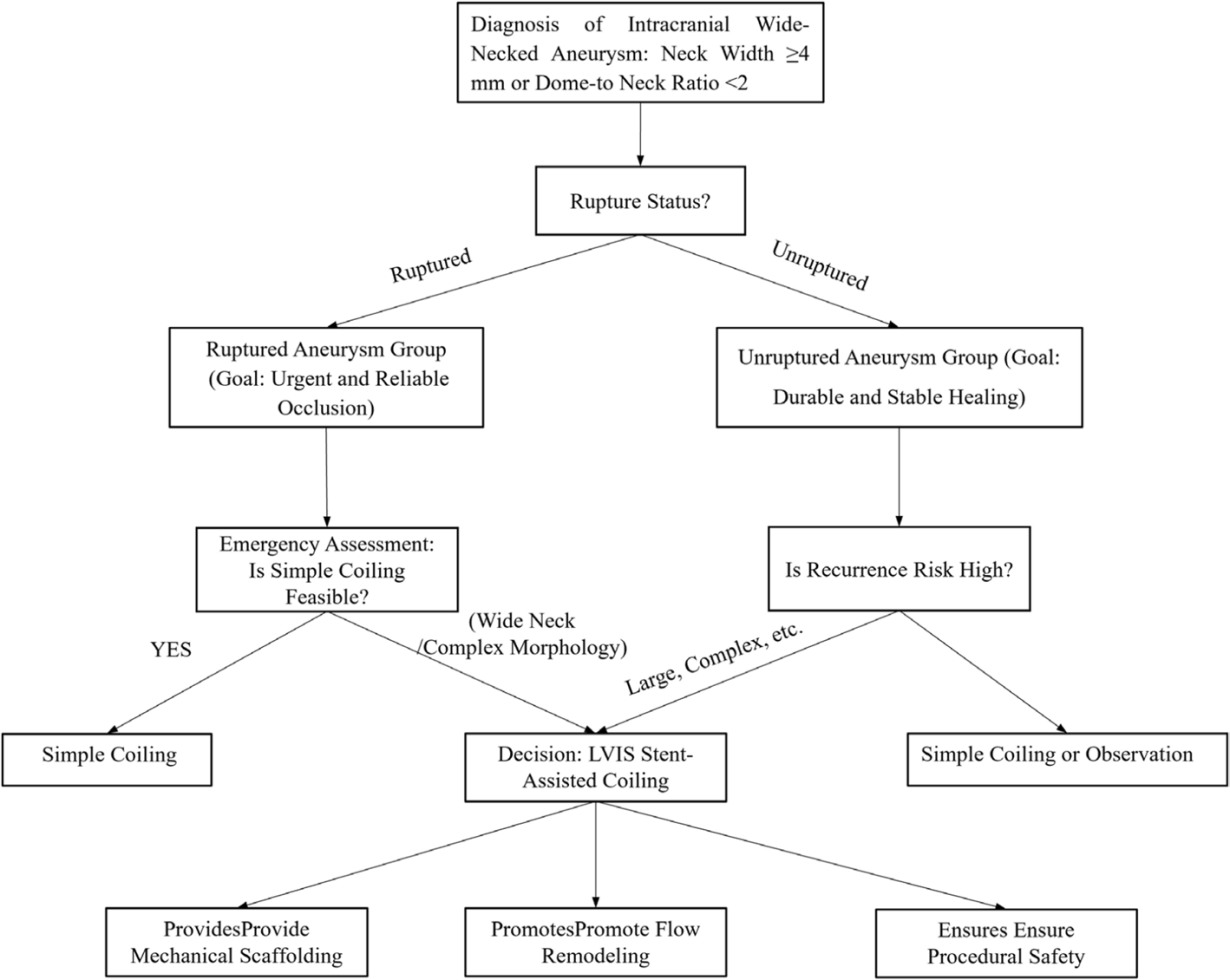
Treatment decision-making algorithm for intracranial wide-necked aneurysms. This flowchart illustrates the structured protocol followed at our center. The initial decision branch is based on aneurysm rupture status

Two skilled attending neuroradiologists independently reevaluated all pre-procedural DSA pictures to guarantee objective and consistent assessment of aneurysm morphology—a crucial factor in treatment selection. Aneurysm type (saccular vs. dissecting/pseudoaneurysm), location (anterior/posterior circulation, bifurcation site), and important morphometric metrics (neck width, dome-to-neck ratio) were all carefully documented. A wide-necked aneurysm was precisely defined as having a neck width ≥ 4 mm or a dome-to-neck ratio < 2, in accordance with the study’s focus. A senior chief physician was consulted in order to establish a final consensus regarding any differences in assessment between the two reviewers. The treatment decision pathway was directly influenced by this thorough re-evaluation procedure (Fig. 1).

#### Aneurysm location and morphology

The topographical and morphological features of the aneurysm were further thoroughly examined in this study based on DSA results. The following were the traits and anatomical distribution:

*Unruptured aneurysms (n = 26)*: Every lesion was found in the anterior circulation. Specifically, the posterior communicating artery segment (C7) accounted for 26.9% (*n* = 7) of cases, while the ophthalmic artery segment (C6) continued to be the most common location, accounting for 69.2% (*n* = 18). One aneurysm (3.8%) was located at the vertebral artery segment V4 in the posterior circulation. 

*Ruptured aneurysms (n = 35)*: The anterior circulation accounted for 94.3% of the ruptured aneurysms (*n* = 33). The posterior connecting artery was the most common location among these, accounting for 82.9% (*n* = 29) of instances and mostly displaying a complicated, irregular, and lobulated morphology. The cavernous sinus section (C4, *n* = 1) and the ophthalmic artery segment (C6, *n* = 2) were additional anterior circulation locations. One saccular aneurysm of the basilar artery and one dissecting aneurysm at the P2 segment of the posterior cerebral artery were the two remaining aneurysms (5.7%) that were discovered in the posterior circulation.

### Endovascular treatment protocol and follow-up

#### Perioperative antithrombotic regimen

For patient care, a consistent antithrombotic strategy was used, with key individualization according to the particular aneurysm status (ruptured or unruptured). In the meantime, the protocol’s pre-procedural verification and post-operative treatment were guided by systematic platelet function testing using light transmission aggregometry (LTA).

## Unruptured aneurysms

Dual antiplatelet therapy (DAPT) with oral aspirin (100 mg/day) and clopidogrel (75 mg/day) for at least 3 days was the pre-procedural treatment. Because of the considerable inter-individual variation in clopidogrel responsiveness, which is especially important in Asian populations because of CYP2C19 polymorphisms, platelet function was evaluated using LTA for every patient prior to the surgery. Only once predetermined inhibition thresholds— ≥ 50% for aggregation induced by arachidonic acid (AA) and ≥ 30% for aggregation induced by adenosine diphosphate (ADP)—were confirmed could the intervention begin [1, 2]. The significance of platelet function testing in modern neurointerventional therapy to reduce perioperative ischemia and hemorrhagic risks was in line with this pre-procedural verification [3, 4]. Additionally, for patients who did not meet these limits, a systematic adjustment strategy was put in place. Specifically, those with inadequate AA inhibition (< 50%) had their aspirin dose increased to 200 mg/day, while clopidogrel hyporesponders (ADP inhibition < 30%) were switched to ticagrelor (60 mg twice daily). Platelet function assessment was repeated after 1 week of adjusted therapy before proceeding. Intraoperatively, systemic heparinization was administered to maintain an activated clotting time at 2–3 times baseline.

## Ruptured aneurysms

**Fig. 2:**
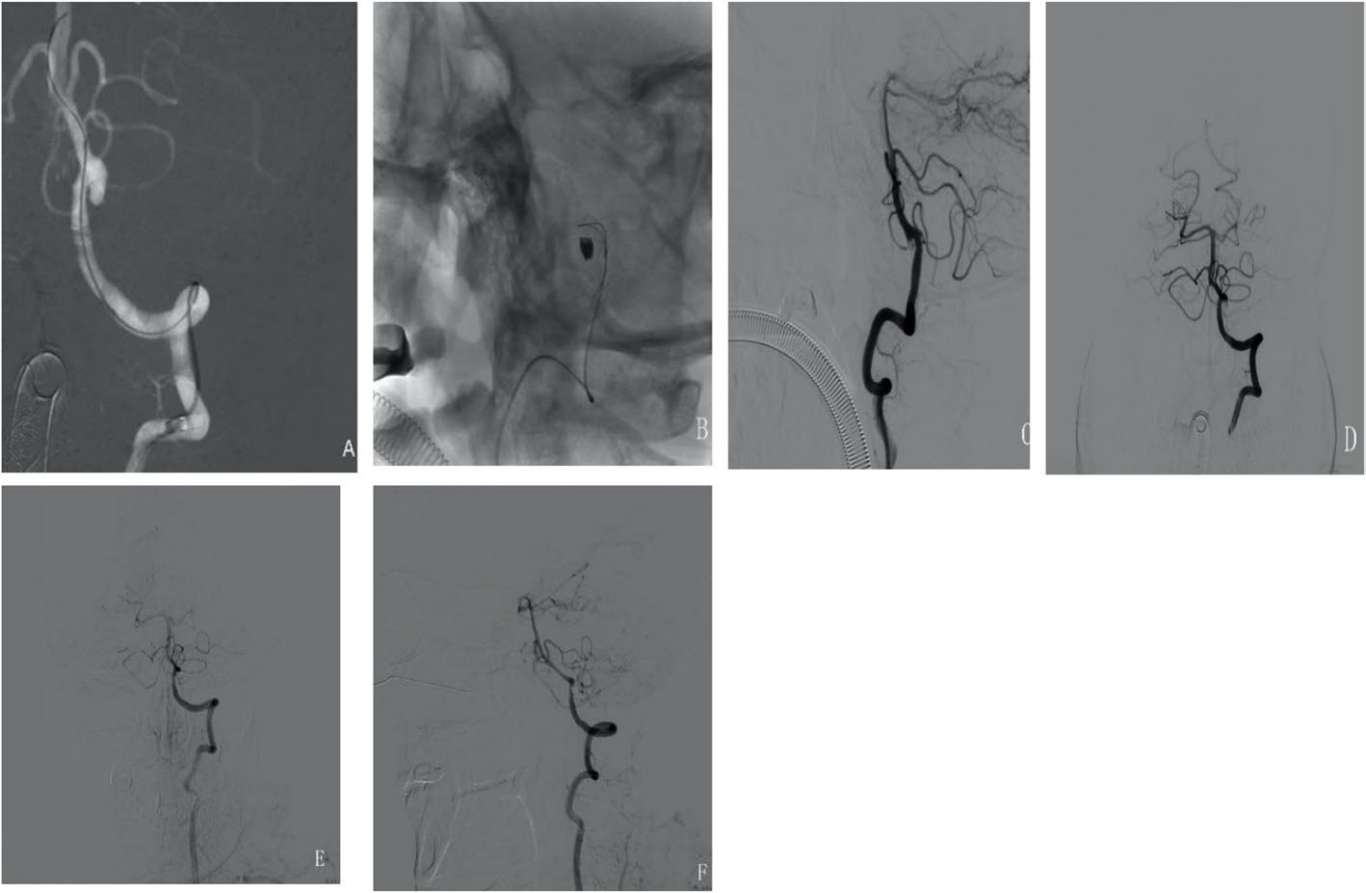
Endovascular treatment of an aneurysm arising from the V4 segment of the vertebral artery using LVIS stent-assisted coiling. **A** Road-map image demonstrating microcatheter navigation into the aneurysm sac before stent deployment (arrows indicate the microcatheter trajectory). **B** After LVIS stent deployment, coil embolization was performed through the stent struts using a microcatheter. Gentle microguidewire massage was performed along the stent segment to improve stent wall apposition. **C** Working projection angiographic image during the procedure demonstrating the deployed stent and progressive coil embolization of the aneurysm. **D** Final angiographic image demonstrating complete aneurysm occlusion (Raymond–Roy class I) with preserved parent artery patency and satisfactory stent expansion. **E** Six-month angiographic follow-up image demonstrating persistent complete aneurysm occlusion with preserved parent artery patency. **F** Additional working projection at 6-month follow-up confirming stable aneurysm occlusion without evidence of recurrence or in-stent stenosis

**Fig. 3:**
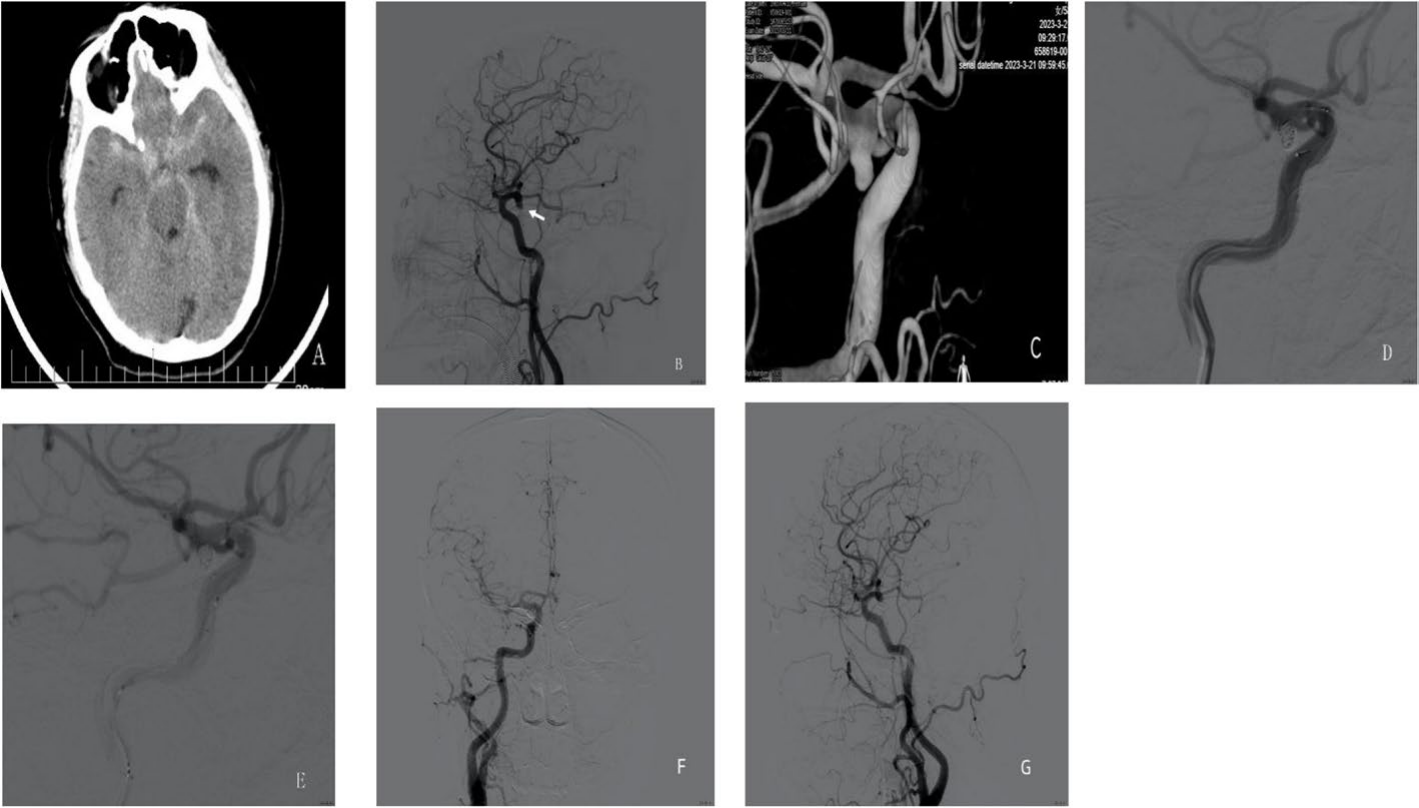
Treatment of the right posterior communicating artery aneurysm with LVIS stent-assisted coiling. **A** Admission non-contrast axial cranial CT demonstrated extensive subarachnoid hemorrhage in the basal cisterns and sylvian fissures (arrowheads), consistent with acute aneurysmal rupture. **B **Right internal carotid artery angiogram (lateral view) revealed a saccular aneurysm (arrow) originating from the posterior communicating artery. **C** Three-dimensional reconstructed angiographic image provided detailed visualization of the aneurysm morphology, neck configuration (asterisk), and its spatial relationship to the parent artery. **D**, **E** Immediate postprocedural angiograms obtained in working projections demonstrated complete aneurysm occlusion according to the Raymond–Roy Occlusion Classification (RROC class I) without residual contrast filling. The deployed LVIS stent adequately covered the aneurysm neck (arrowheads) with preserved patency of the parent vessel. **F**, **G** Six-month follow-up angiograms in anteroposterior (**F**) and lateral (**G**) views confirmed maintained complete occlusion (RROC class I) without evidence of aneurysm recurrence or in-stent stenosis

Pre-operative oral antiplatelet loading was not administered in any patient (0/35, 0%) in the ruptured aneurysm group, in accordance with previous reports recommending caution with antiplatelet therapy in the acute phase of subarachnoid hemorrhage to minimize the risk of hemorrhagic complications [5, 6]. Following deployment of the LVIS stent, a 0.4 mg bolus of tirofiban (8 mL of solution at 0.05 mg/mL) was administered intra-arterially via the guiding catheter as a slow push over 1–2 min to prevent acute thrombus formation associated with intracranial stenting. Postoperatively, a continuous intravenous infusion of tirofiban was initiated at a rate of 0.1 μg/kg/min and maintained for 24–48 h. The transition to oral DAPT was achieved with aspirin (100 mg/day) and clopidogrel (75 mg/day) on postoperative day 1, with the tirofiban infusion continued for a mandatory overlap period of at least 24 h. Platelet function was subsequently verified using LTA to ensure adequate P2Y12 inhibition during this critical transition period [7, 8]. In the majority of cases (33/35), this protocol was sufficient without intraprocedural thrombotic events. In the remaining two cases, however, acute intraprocedural thrombus formation was detected angiographically. Of note, the same 0.4 mg dose was used as the rescue bolus rather than a higher dose, because a higher bolus might increase the risk of rebleeding in the setting of acute subarachnoid hemorrhage. In these cases, rescue therapy was implemented as follows: a rescue bolus of 0.4 mg tirofiban (8 mL of solution at 0.05 mg/mL) was administered intra-arterially via the intermediate catheter as a slow push over 1–2 min. If the thrombus persisted, a microguidewire was carefully advanced across the thrombus under fluoroscopic guidance over approximately 1–2 min, followed by navigation of a microcatheter through the thrombus. Subsequently, an additional 1–2-mL of tirofiban solution (0.05 mg/mL, i.e., 0.05–0.10 mg of tirofiban) was slowly injected via the microcatheter over 1–2 min to facilitate thrombus dissolution. Following these intraprocedural measures, postoperative intravenous infusion of tirofiban was initiated at a rate of 0.1 μg/kg/min for 24–48 h, and the infusion rate was adjusted based on clinical symptoms and imaging findings (including head CT and head CTA), with the possibility of increasing up to 0.2 μg/kg/min if clinically indicated (Figs. 2 and 3).

## Post-operative maintenance for all patients

All patients were given maintenance therapy of oral DAPT (aspirin 100 mg/day and clopidogrel 75 mg/day) for a minimum of 6 months. Subsequent decisions regarding therapy de-escalation or DAPT extension were individualized, considering the follow-up angiography, implanted device type, serial LTA results, and a continuous assessment of ischemic versus hemorrhagic risk comprehensively.

Management of patients requiring post-procedural external ventricular drainage (EVD).

In this cohort, 4 out of 35 patients (11.4%) with ruptured aneurysms were observed with persistent depressed consciousness or radiographic progression on follow-up CT scans. Therefore, this part of patients developed symptomatic hydrocephalus after the endovascular procedure. These patients subsequently underwent EVD.

At the time of EVD placement, all the 4 patients were already receiving post-operative DAPT regimen (clopidogrel and aspirin) as outlined above, which was considered essential for stent protection. Critically, the DAPT regimen was not adjusted or interrupted for the EVD procedure.

### Stent-assisted coiling technique

All procedures were performed under general anesthesia. An 8F introducer sheath was placed via a transfemoral approach, followed by the navigation of a 6 F guiding catheter to the target parent artery. Three-dimensional rotational digital subtraction angiography (3D-DSA) was used to evaluate aneurysm morphology, neck width, and parent vessel anatomy.

Using the stent-assisted coiling technique, an Echelon-10 microcatheter (ev3/Covidien, USA) was first navigated into the aneurysm sac over a microguidewire. Subsequently, an appropriately sized LVIS stent (MicroVention, USA) was partially deployed across the aneurysm neck to provide initial neck coverage while maintaining access to the aneurysm sac. Under this semi-deployment state, detachable coils were sequentially inserted into the aneurysm sac to achieve satisfactory packing density.

After satisfactory coil embolization was achieved, the LVIS stent was fully deployed to complete reconstruction of the parent vessel and stabilize the coil mass at the aneurysm neck. Final angiographic assessment was then performed to evaluate stent–vessel wall apposition and to confirm the absence of in-stent thrombosis, branch vessel occlusion, or coil protrusion into the parent artery.

### Outcome assessment and follow-up protocol

The RROC was used to assess the immediate angiographic data. Patients were categorized as Class I (total occlusion), Class II (residual neck), or Class III (residual aneurysm) based on this. In the meantime, patients were monitored for 6 to 24 months using standardized tests such as:

- Procedure-related complications documentation;
- Serial imaging evaluation (DSA/MRA);
- Neurological status monitoring.

At 6 months, patients had a functional outcome assessment using the modified Rankin Scale (mRS). Patients were therefore classified as either favorable (mRS 0–2, functional independence) or unfavorable (mRS 3–5, functional dependence). Evaluation of follow-up DSA:

- Aneurysm occlusion status;
- Parent artery patency;
- Stent stability;
- Complications including hemorrhagic/ischemic events and recurrence.

### CFD data analysis

To investigate the hemodynamic mechanisms associated with the treatment, CFD analysis was conducted on a consecutively enrolled subset of 14 patients who had both baseline and follow-up angiographic data available. This cohort comprised 4 patients with ruptured and 10 with unruptured aneurysms. All patients in this subset exhibited favorable immediate angiographic outcomes, defined as RROC I or II. Specifically, within the ruptured subgroup, 3 cases achieved complete occlusion (RROC I), and 1 case had a neck remnant (RROC II). In the unruptured subgroup, 9 aneurysms were completely occluded (RROC I) and 1 showed a neck remnant (RROC II).

Pre- and post-implantation models were reconstructed to quantify changes in key parameters: relative inflow, relative flow volume (RFV), and WSS. Continuous data are presented as mean ± standard deviation. Paired-sample *t*-tests were employed to compare pre- and post-operative parameters, with a *P*-value < 0.05 considered statistically significant (SPSS 26.0).

## Results

### Study cohort and immediate angiographic outcomes

This study was conducted based on the enrollment of a cohort involving 35 with ruptured aneurysms and 26 with unruptured aneurysms. According to the immediate post-operative DSA, in the ruptured aneurysm group, 28 cases (80%) achieved RROC class I, and 7 cases (20%) were class II; while in the unruptured aneurysm group, 22 cases (84.6%) had RROC class I, and 4 cases (15.4%) were class II.

### Angiographic follow-up and evolution

The overall angiographic follow-up rate was 44.3% (27/61), with completion rates of 37.1% (13/35) and 53.8% (14/26) in the ruptured and unruptured aneurysm groups, respectively. At the 6- to 12-month follow-up, among the ruptured aneurysms available for imaging, one of the two initially class II lesions improved to complete occlusion (class I). In the unruptured cohort, one of the two initially class II aneurysms altered to class I, while the other remained class II. All aneurysms that were initially completely occluded (class I) in both groups demonstrated sustained stable occlusion.

### Procedure-related complications and safety profile

Regarding intraoperative complications, two cases of intraprocedural thrombosis occurred in the ruptured aneurysm group (2/35, 5.7%) and were successfully managed using the rescue protocol described in the “Methods” section, without permanent neurological deficits.

Following LVIS stent deployment, incomplete stent apposition (ISA) was observed in 6 of 61 patients (9.8%), including four cases in the ruptured aneurysm group and two cases in the unruptured aneurysm group. All ISA cases were identified intraoperatively using standard digital subtraction angiography. Gentle microguidewire massage was attempted in all cases to improve stent wall apposition, without the implementation of balloon angioplasty.

Post-operatively, acute hydrocephalus developed in 4 of the 35 patients with ruptured aneurysms (11.4%), necessitating EVD. Notably, none of these patients experienced subsequent re-bleeding following the EVD procedure.

One patient in the unruptured aneurysm group experienced a fatal complication associated with delayed in-stent thrombosis. In this case, an LVIS stent was deployed across the aneurysm neck during stent-assisted coiling. Mild-to-moderate ISA was noted right after deployment. Although stent wall apposition was improved by gentle microguidewire massage, full correction was not attained.

In accordance with our institutional procedure, the patient received routine preoperative dual antiplatelet medication and antithrombotic treatment. The patient experienced new neurological complaints in the early postoperative period, and a repeat angiography at that time revealed no major large-vessel obstruction. Over the next 3 to 5 days, however, neurological state gradually declined.

In-stent thrombosis with parent artery blockage was discovered by subsequent angiography. Despite attempts at endovascular rescue therapy, such as mechanical thrombectomy, adequate recanalization was not achieved. Despite receiving medical care, the patient eventually passed away after developing a secondary cerebral hemorrhage.

As a result, the overall procedure-related mortality and morbidity (mRS 3–5 at discharge) rates were 1.6% (1/61) and 16.4% (10/61), respectively, with the ruptured aneurysm group accounting for all morbidity cases.

### Functional outcomes and recovery

Functional outcomes at discharge and during follow-up were detailed below by aneurysm status.

#### Unruptured aneurysm group (n = 26)

At discharge, 96.2% (25/26) of patients achieved favorable outcomes (mRS 0–1), and one patient (3.8%) died in-hospital due to stent thrombosis. At 3–6 months, all surviving patients (25/25) maintained favorable outcomes (mRS 0–1).

#### Ruptured aneurysm group (n = 35)

At discharge, 57.1% (20/35) had favorable outcomes (mRS 0–1), 28.6% (10/35) sustained moderate-to-severe disability (mRS 3–5), and no patient had died (mRS 6).

Significant neurological recovery was observed in this group during follow-up. The rate of favorable outcomes (mRS 0–1) increased from 57.1% at discharge to 77.1% (27/35) at 3–6 months. In contrast, the rate of significant morbidity (mRS 3–5) decreased from 28.6% to 14.3% (5/35) (Table 2). No deaths occurred during follow-up.

**Table 2:** Functional recovery in patients with ruptured aneurysms from discharge to 3–6 month follow-up (*n* = 35)

mRS category	**At discharge (*n*%)**	**At 3–6 month follow-up**	Absolute change
Favorable outcome (0–1)	20 (57.1%)	27 (77.1%)	+ 20%
Slight disability (2)	5 (14.3%)	3 (8.6%)	− 5.7%
Significant morbidity (3–5)	10 (28.6%)	5 (14.3%)	− 14.3%
**Death (6)**	0	0	0

## Summary

Both groups were followed up with high rates of favorable functional outcomes. The ruptured cohort exhibited marked recovery from discharge to 3–6 months, with a pronounced decline in moderate-to-severe disability.

### Hemodynamic analysis

Hemodynamic analysis indicated that LVIS stent implantation was associated with significant alterations in intra-aneurysmal flow patterns (Table 3). Post-operatively, all core parameters were remarkably reduced compared to the pre-operative baselines. Specifically, the relative inflow decreased from 0.17 ± 0.11 to 0.07 ± 0.04, the proportion of aneurysm volume with high relative flow (RFV > 0.1%) decreased from 65.8 ± 20.1% to 9.2 ± 8.7%, and the mean WSS decreased from 4.28 ± 1.49 Pa to 1.31 ± 0.51 Pa (all < 0.001). These altered parameters persisted at the 6-month follow-up, remaining significantly different from pre-operative values (all *P* < 0.001).

**Table 3:** Comparison of core hemodynamic parameters before and after LVIS stent implantation (x ± s)

Parameter	Pre-operative	Post-operative	6-month follow-up	vs pre-operative		6-Month follow-up vs pre-operative	
				*t*-value	*P*-value	*t*-value	*P*-value
Relative Inflow	0.17 ± 0.11	0.07 ± 0.04	0.09 ± 0.06	5.12	< 0.001	4.65	< 0.001
Aneurysm RFV > 0.1 (%)	65.8 ± 20.1	9.2 ± 8.7	11.5 ± 12.3	11.85	< 0.001	10.28	< 0.001
Aneurysm WSS (Pa)	4.28 ± 1.49	1.31 ± 0.51	1.35 ± 0.69	9.41	< 0.001	8.17	< 0.001

Table note: *RFV*, relative flow volume; and *WSS*, wall shear stress. In the comparison of both post-operative and 6-month follow-up values with pre-operative values, all parameters showed highly statistically significant differences (*P*< 0.001).

## Discussion

Focusing on 61 patients with wide-necked intracranial aneurysms, this single-center, retrospective analysis evaluated the safety, short-term efficacy, and hemodynamic effects of LVIS stent-assisted coiling over a 1-year period. This study was conducted by integrating clinical and hemodynamic data, providing observational insights into the performance of this device. It is important to frame the findings within the context of the study’s design.

### Clinical outcomes

The observed angiographic outcomes in our cohort were encouraging. Immediate complete occlusion (RROC I) was achieved in 80.0% of ruptured and 84.6% of unruptured aneurysms. Among patients who underwent 6–12 month follow-up angiography (44.3%, 27/61), there were relatively high rates of stable or improved complete occlusion, suggesting progressive healing. The flow-disruptive mechanism associated with braided stents is compatible with this “delayed occlusion” effect. In comparison to other historical benchmarks established for stent-assisted coiling procedures, the occlusion rates found in our group seemed favorable [9]. However, considering the considerable variation in patient selection, aneurysm features, and operative protocols, any direct inter-study comparisons must be evaluated cautiously [9].

### Safety profile and complication management

The safety study of our retrospective cohort showed that aneurysm rupture status had a significant impact on the risk profile. The morbidity rate (mRS 3–5 at discharge) was 16.4% (10/61), and the overall procedure-related mortality was 1.6% (1/61). However, a clear intergroup contrast is concealed by this aggregate figure. A significant burden of procedure-attributable morbidity (28.6%, 10/35) was found in the ruptured aneurysm group (*n* = 35), indicating the superimposition of procedural risk on a brain damaged by the initial hemorrhage. On the other hand, 96.2% (25/26) of the unruptured group (*n* = 26) were discharged with an excellent outcome (mRS 0–1), indicating low functional morbidity. Despite this, this group was responsible for the lone death from fatal in-stent thrombosis.

#### Thromboembolic complications and management

Of the total patients, 4.9% (3/61) experienced symptomatic thromboembolic events. This incidence comprised one fatal in-stent thrombosis in an unruptured instance and two intraprocedural thromboses in the ruptured aneurysm group that were successfully treated with rescue therapy.This number was consistent with the 4.6% cumulative morbidity and fatality rate seen in a prospective, multicenter LEPI study that assessed LVIS devices in an actual scenario [10]. Additionally, this rate was within the wide range predicted for stent-assisted coiling methods generally [11–14]. Notably, the one fatal in-stent thrombosis in an unruptured patient highlights the fact that, despite being rare, catastrophic thromboembolic consequences are still a real procedure-related risk, even in patients that are adequately treated.

#### Customized antithrombotic techniques: observational experience with institutional protocols

The antithrombotic regimens discussed here are customized institutional measures designed to balance hemorrhagic and ischemic risks. Rather than being evidence-based recommendations, these schemes should be seen as observational experiences from a particular population.

This study developed a standardized pre-operative DAPT regimen based on platelet function testing (LTA) for unruptured aneurysms. The fatal thrombosis in this subgroup occurred in a patient with identified inadequate platelet inhibition despite pre-procedural testing. It highlights the role of inter-individual variability in drug response and the limitations of platelet function testing [1]. Therefore, even “adequate” laboratory parameters do not eliminate thrombotic risk completely.

In patients with ruptured aneurysms undergoing stent-assisted coiling, the optimal antiplatelet regimen remains a clinical challenge due to the competing risks of thromboembolism and rebleeding [5–7]. In our cohort, we avoided pre-operative oral antiplatelet loading in all ruptured cases (0/35, 0%) to minimize the risk of hemorrhagic complications in the acute phase of subarachnoid hemorrhage, consistent with previous reports recommending caution with antiplatelet therapy in this setting [5, 6]. Instead, we adopted an intraoperative intra-arterial tirofiban protocol. Of note, the same 0.4 mg bolus dose was used for both routine prevention and as the initial rescue bolus in the two cases with thrombotic complications. We deliberately did not escalate the bolus dose because a higher dose might increase the risk of rebleeding. To achieve an enhanced antiplatelet effect without increasing the bolus dose, we employed a stepwise escalation strategy: (1) delivering the rescue bolus via the intermediate catheter (closer to the thrombus); (2) targeted local delivery of an additional 1–2 mL (0.05–0.10 mg) via a microcatheter advanced through the thrombus; and (3) increasing the postoperative intravenous infusion rate from 0.1 μg/kg/min up to 0.2 μg/kg/min based on clinical symptoms and imaging findings (including head CT and head CTA). This approach balanced the need for additional antiplatelet effect against the risk of rebleeding.

The use of glycoprotein IIb/IIIa inhibitors in this rescue context is supported by their documented feasibility in managing acute thrombosis during neurovascular procedures [7]. In our series, the thromboembolic complication rate in ruptured aneurysms (2/35, 5.7%) compared favorably with the pooled rate of 11.2% (95% CI 9.2%–13.6%) reported in a systematic review by Ryu et al. [7], suggesting that our modified protocol may be effective. Nevertheless, the occurrence of two intraprocedural thrombotic events indicates that even with aggressive antiplatelet therapy, thrombotic complications cannot be completely eliminated in complex ruptured aneurysms.

This protocol was further tested in the subset of ruptured patients (*n* = 4) who required EVD. The antithrombotic regimen (tirofiban infusion bridging to DAPT) was not interrupted for EVD placement or management. Meanwhile, no EVD-related re-bleeding occurred in this small subgroup. This experience, while informative, pertains to a limited sample size. So far, it remains an area of ongoing investigation and debate concerning the optimal management of antiplatelet therapy in stent-assisted coiling, particularly regarding agent selection, monitoring, and duration [8].

#### ISA: assessment, value of novel technology, and management considerations

ISA is a technical factor of interest in stent-assisted coiling. In this study, the incidence of ISA was 9.8% (6/61) based on conventional DSA. Notably, the single case of fatal in-stent thrombosis in one patient with unruptured aneurysm was associated with angiographically identified ISA, suggesting a potential clinical link. This finding was comparable to results from a large real-world LEPI study. That LEPI study, which evaluated the safety of LVIS/LVIS Jr devices, despite the report of a low stent deployment failure rate (2.6%), also emphasized the association between technical success and long-term safety [10]. Consistent with the observation in our ISA cases, that study highlighted that achieving optimal device apposition intraoperatively is fundamental for complication prevention.

ISA may be caused by a number of anatomical and technical causes. Particularly in distant intracranial veins, vessel tortuosity and curvature at the aneurysm neck may restrict full stent expansion and wall apposition. Furthermore, insufficient adaptation upon deployment may arise from a mismatch between the stent diameter and the caliber of the parent channel. Although the LVIS stent’s braided design is beneficial for covering the neck, it may occasionally increase the risk of localized malapposition in highly curved arterial segments.

However, when evaluating ISA, traditional two-dimensional angiography might not be adequate. A more accurate assessment technique is offered by newly developed high-resolution three-dimensional fusion imaging. For instance, a specific investigation on the assessment of LVIS stent apposition utilizing this technology was carried out by Kato et al. (2024). They reported detection rates of 47.5% for edge malapposition and 27.5% for the crescent sign (trunk malapposition), which were considerably higher than results from traditional angiography and showed strong inter-rater reliability [15]. This striking disparity strongly points to a widespread risk in normal practice of failing to notice mild malapposition. This method makes it possible to clearly see the spatial relationship between the stent structure and the vascular wall by combining cone-beam CT with DSA pictures. Its benefit is that it gives the operator exact, three-dimensional, and almost real-time feedback. It might be an important tool for maximizing stent deployment and getting the “first-time best” result.

From a management perspective, risk–benefit considerations are required regarding strategies for significant ISA detected or suspected intraoperatively. Integrating literature and technological advances, for persistent and significant malapposition, concurrent intraoperative balloon angioplasty has been reported as a potential corrective option to improve stent–vessel wall apposition, reduce flow disturbance, and lower potential thrombogenic risk [16]. However, in the present study, only microguidewire massage was routinely performed to improve stent apposition. In most cases, microguidewire manipulation resulted in satisfactory improvement in stent–vessel wall apposition on final angiographic assessment, without evidence of persistent significant malapposition. Balloon angioplasty or additional stent placement was not attempted because the ISA observed intraoperatively was generally considered mild and did not result in significant flow compromise. Moreover, previous reports have indicated that aggressive corrective maneuvers, including balloon dilation within an intracranial stent, may carry potential risks such as vessel injury, hemorrhagic complications, or device migration. Therefore, a relatively conservative strategy was adopted in our center.

Importantly, whether modifying antiplatelet regimens (particularly balancing hemorrhagic and ischemic risks in ruptured aneurysms) [5–7] or using remedial techniques (e.g., balloon angioplasty), specific intraoperative assessment and multifactorial consideration should be given priority in the application of any measure. The previously described LEPI study also suggested the variety of antithrombotic treatment in real-world situations, where some patients did not receive standard pre-treatment, which may have affected the clinical results [10]. As a result, the ISA seen in this study and its management experience should be viewed as real-world observations under particular technological circumstances and patient demographics. The majority of patients had progressive stent adaptation, according to follow-up angiography in the cases that were available, and there was no indication of ongoing, clinically significant malapposition. To investigate and elucidate the best course of action, more prospective studies with high-resolution imaging evaluation ought to be carried out in the future

### Hemodynamic effects and clinical implications

Beyond its function as a mechanical scaffold, stent-assisted coiling’s ability to alter intra-aneurysmal hemodynamics—a process connected to thrombosis and recurrence prevention—may contribute to its therapeutic efficacy [17]. The flow-modifying effects of the LVIS stent were measured in this study using CFD analysis.

After LVIS-assisted coiling, our observational analysis showed changes in hemodynamic parameters. Relative inflow, the percentage of aneurysm volume with high RFV (> 0.1), and mean WSS all decreased after surgery. At the 6-month follow-up, however, these changes seemed to be stable. Based on these factors, an intra-aneurysmal environment that is less favorable to blood flow activity may be caused by an LVIS stent. This background draws attention to a possible market for products like the LVIS stent in certain situations involving ruptured aneurysms.

The braided design and metal coverage (~ 23%) of the LVIS stent, which is more than that of laser-cut stents (such as Enterprise), were consistent with the hemodynamic changes observed in our investigation. A single LVIS stent was found to reduce intra-aneurysmal flow more than two Enterprise stents in a previous comparative CFD analysis, however the effect was less noticeable than with a dedicated Pipeline Embolization Device (PED) [18]. There is clinical interest in this intermediate hemodynamic profile. For complex unruptured aneurysms, delicate flow diverters (like PED) are used because of their strong flow disruption [19, 20]. However, the related thromboembolic risk and the requirement for rigorous antiplatelet medication, which presents a major barrier in the acute hemorrhagic phase, may limit their usage in acutely ruptured cases [21, 22]. Thus, the intermediate flow-diversion capacity of the LVIS stent may be a unique treatment option for ruptured aneurysm, as shown by our observational data; nevertheless, the risk of intense antiplatelet therapy must be weighed against the benefit of flow modification.

## Conclusions

In this observational series, LVIS stent-assisted coiling may be linked to positive mid-term stability and high initial angiographic occlusion rates in wide-necked intracranial aneurysms, with CFD analysis confirming significant post-procedural hemodynamic alteration.

There is a clear safety dichotomy, nevertheless, as evidenced by the ruptured aneurysm cohort’s significant morbidity, underscoring the additional danger of acute intervention. Technical success seems to be critical, especially for ideal stent apposition.

The viability of the LVIS stent for this indication is supported by observational data presented in this paper. It raises questions, especially about its possible function in situations where intense antiplatelet therapy is an issue. These results clearly highlight the necessity for additional prospective, controlled research to conclusively determine the relative efficacy and safety of this therapy approach.

### Limitations

To properly interpret the results, it is necessary to acknowledge a number of significant limitations of this study.

First, definite causal inferences would be impossible due to the retrospective, single-center methodology, which would naturally restrict the generalizability of our findings. Although internally consistent, the sequential patient cohort represented a single institution’s experience.

Second, a consistent interpretation of the results were made more difficult by the clinical heterogeneity of the group, which included both ruptured and unruptured aneurysms with different sites and shapes. Given the significantly varied risk profiles between the two groupings, a cautious, stratified interpretation of pooled results is therefore required.

 Third, selection bias may be introduced by the inadequate angiographic follow-up rate (44.3%). It could be explained by the possibility that patients who came back for follow-up reflect a subset with better results, perhaps inflating the long-term effectiveness.

Fourth, a selection of patients underwent the exploratory CFD analysis. Although significant hemodynamic changes were noted, this investigation lacked the power to directly link particular flow characteristics to clinical outcomes like recurrence.

Lastly, the evaluation of stent apposition depended on traditional angiography, which is less sensitive than newly developed high-resolution three-dimensional fusion imaging for identifying minute malapposition [15]. 

## Data Availability

Data Availability Statement The clinical and imaging datasets generated and analyzed during this retrospective study are not publicly available due to patient privacy and ethical restrictions stipulated by the Institutional Review Board of Tangshan Workers’ Hospital. However, de-identified data supporting the findings of this study may be made available from the corresponding author (Xu Xiang) upon reasonable request, subject to review and approval by the ethics committee.

## Abbreviations

RROC: Raymond-Roy Occlusion Classification
mRS: modified Rankin Scale
DSA: Digital subtraction angiography
CFD: Computational fluid dynamics
WSS: Wall shear stress
LVIS: Low-profile visualized intraluminal support
SAC: Stent-assisted coiling
BAC: Balloon-assisted coiling
CT: Computed tomography
MRA: Magnetic resonance angiography
LTA: Light transmission aggregometry
DAPT: Dual antiplatelet therapy
ADP: Adenosine diphosphate
EVD: External ventricular drainage
3D-DSA: Three-dimensional rotational digital subtraction angiography
ISA: Incomplete stent apposition
